# Implementation of National Emergency X-Radiography Utilization Study (NEXUS) Criteria in Pediatrics: A Systematic Review

**DOI:** 10.7759/cureus.30065

**Published:** 2022-10-08

**Authors:** Chukwuyem Ekhator, Ijeoma Nwankwo, Akito Nicol

**Affiliations:** 1 Neuro-Oncology, New York Institute of Technology, College of Osteopathic Medicine, Old Westbury, USA; 2 Research, California Institute of Behavioral Neurosciences & Psychology, Fairfield, USA; 3 Radiology, New York Institute of Technology, College of Osteopathic Medicine, Old Westbury, USA

**Keywords:** ct scan, cervical spine, radiology, nexus criteria, pediatrics

## Abstract

Since its introduction in 1992, the National Emergency X-Radiography Utilization Study (NEXUS) criteria have been used in trauma to decide whether a patient requires radiographic imaging. The tool is important in reducing radiation exposure. However, applying the NEXUS criteria for cervical spine imaging in pediatric patients is poorly supported compared to their use in adults. The objective of this review was to examine the effectiveness of using the NEXUS criteria in the diagnostic management of pediatric cervical spine injuries (CSI). The following databases were searched for studies focused on applying the NEXUS criteria for CSI in pediatric patients: Cochrane, PubMed, Google Scholar, EMBASE, ELSEVIER, and ScienceDirect. Additional studies were found through reference lists of primary sources and previous systematic and meta-analyses.

The search focused on randomized controlled trials (RCTs), cohort studies, retrospective studies, prospective studies, and other uncontrolled trials published from 2000 to 2022. There were seven included studies with a total of 4502 pediatric patients. Five of the included studies were retrospective studies, while the remaining were prospective and case studies. Our results show that the sensitivity ranged from 43% to 100%, while the specificity ranged from 12.93% to 96%. The sensitivity increased with age, with those under the age of two or under the age of eight reporting poorer outcomes than the older pediatric patients. One study also shows that the proportion of patients undergoing cervical spine CT increased from 18% to 61% in the initial period before the implementation of clearance guidelines. The implementation of guidelines led to a 23% decrease in CT scans clearable by NEXUS criteria after 12 months. One of the studies reported that NEXUS criteria were a cost-effective option when used along with X-rays and CT. Overall, the studies do not strongly support the application of the NEXUS criteria to image pediatric patients for CSI. In conclusion, there is weak support in the literature for applying the NEXUS criteria in determining the need for cervical spine imaging in pediatric trauma patients. The practice and research implications of the findings are also discussed.

## Introduction and background

Cervical spine injuries (CSI), mainly caused by falls and traffic accidents, are associated with damage to other organs, reduced quality of life, increased morbidity, and increased mortality rates [1]. The detection of cervical spine stability is challenging when performed during injury and reduced consciousness [2]. This implies that it is important that the selected radiography and selection criteria are critical in improving diagnosis. In addition, risk stratification strategies are important in limiting interventions that may be harmful to patients who may have spine injuries [3].

The National Emergency X-Radiography Utilization Study (NEXUS) criteria comprise a decision tool that has the potential to improve processes and imaging outcomes. The NEXUS criteria are useful in deciding whether or not trauma patients require cervical spine imaging. In the original study, the NEXUS criteria were found to have a sensitivity of 99.6% (98.6%-100%) at a 95% confidence interval [4]. The NEXUS criteria are defined as the presence of at least one of the following: altered consciousness, midline cervical spine tenderness to palpation, distracting injury, intoxication, or neurologic deficit [5]. The five criteria are collectively referred to as the NEXUS low-risk criteria (NLC) [6]. Cervical spine imaging is indicated if any of the five criteria are met. The validation of the criteria in the original study has led to its widespread use despite concerns about its effectiveness in some groups. Specifically, concerns have been raised about the sensitivity of the criteria in elderly and pediatric patients [7,8].

The use of the NEXUS criteria in pediatric patients has not been strongly supported in the literature. The criteria were developed using a study group of 34,069 patients, with only 2.5% aged eight years or younger [9]. Additionally, only 30 participants in the original study had CSI. The numbers in the initial study do not reflect true CSI patterns, thus reducing the validity of applying the NEXUS criteria for children. While studies such as the Pediatric Emergency Care Applied Research Network (PECARN) study have sought to address the small pediatric population problem in NEXUS, they lack perspective validation [3,10]. These factors emphasize the importance of evaluating the validity of the NEXUS criteria in pediatric patients in CSI assessment. The issues with the development of the criteria have legitimately raised the urgency and importance of reassessing the validity, accuracy, and effectiveness of NEXUS in pediatric patients.

While limited, researchers have increasingly sought to explore the validity of the NEXUS criteria in pediatric patients. Most current literature has focused on blunt head trauma, with CSI receiving limited attention. Few studies have confirmed the validity of NEXUS criteria in pediatric patients, with most being cautious about its application in children [8,11]. In their study, Viccellio et al. cautiously endorsed using the NEXUS criteria for pediatric patients aged between 0 and 9 years [11]. According to Kos et al., the NEXUS criteria should only be used in aiding clinical gestalt since it is yet to be validated in pediatric trauma [8]. The inability of infants and toddlers to fully express themselves makes assessment of the validity of the NEXUS criteria complicated.

The unique anatomic features of children, such as flexible spines and large head sizes, also predispose the group to spinal cord injuries [12]. While uncommon among pediatric patients (1%-10%), spinal trauma, including CSI, significantly contributes to morbidity and mortality in this group [12]. CSI is difficult to diagnose in pediatric patients compared to adults as they tend to occur in different locations [13]. Improved knowledge in applying the NEXUS criteria to support decisions on obtaining CT, X-ray, or MRI is necessary for pediatric trauma care.

The evidence for the effectiveness of the NEXUS criteria in adults demonstrates their potential use in pediatric patients to diagnose CSI and other spinal trauma. While the NEXUS criteria have been applied to pediatric patients, the small populations of infants and toddlers in the studies imply that the validity of the criteria remains unknown [14]. There is a gap in the current literature with respect to incorporating infants, toddlers, and other children in the validation of the NEXUS criteria. This leads to uncertainty when applying the criteria in the group, thus increasing the risk of unnecessary exposure of pediatric patients to radiation.

Given the associated risks, the decision to proceed with cervical spine imaging is critical and ought to be made after adequate consideration. The potential of the NEXUS criteria to improve decision-making in pediatric imaging needs further evaluation. The objective of the current study is to conduct a literature review on the effectiveness of the NEXUS criteria as a selection tool for imaging pediatric patients with CSI.

## Review

Methods 

This study examines the application of the NEXUS criteria in pediatric patients using the literature analysis approach. The systematic review explores the effectiveness and efficacy of NEXUS criteria for pediatric patients with CSI. The study will also consider the application of NEXUS in other traumas in pediatric patients and comparative performance relative to other assessment tools. A comprehensive literature review is necessary to realize the objectives of the study. This section presents the search strategy, inclusion/exclusion criteria, outcome measures, quality evaluation approach, and the data extraction and analysis methods used in the study.

Search strategy

The current literature review examined prospective and retrospective studies on applying the NEXUS criteria in evaluating pediatric patients for CSI and other spinal trauma. The electronic databases that were used to search articles for inclusion are Cochrane, PubMed, Google Scholar, EMBASE, Elsevier, and Science Direct. The search focused on randomized controlled trials (RCTs), cohort studies, retrospective studies, prospective studies, and other uncontrolled trials published from 2000 to 2022. The year 2000 was selected as it was when the fifth criterion was added. Other than the database search, the reference lists and systematic reviews consistent with the topic of the present study were considered for additional studies.

A robust search strategy was used to identify English publications that addressed the research topic of the study. The search strategy used English language and medical subject headings (MeSH) terms to locate the most appropriate articles for inclusion. The keywords that were used in the search include "NEXUS criteria", "NEXUS sensitivity", "NEXUS selectivity", "pediatric", "child", "cervical spine injury", "c-spine imaging", "CSI", "spinal trauma", and "decision support technique". The keywords were used in different combinations to identify the studies that fit the research topic of the current systematic review.

Inclusion and exclusion criteria

The inclusion and exclusion criteria are outlined in Table 1 below.

**Table 1:** Inclusion and exclusion criteria

Inclusion Criteria	Exclusion Criteria
Articles published between 2012 and 2022	Articles that discuss NEXUS criteria as the key assessment technique
Articles where treatment groups used NEXUS as the assessment tool	Articles where adults were the treatment group
Articles that focused on pediatric patients (Aged between 0 and 18 years)	Articles that lack controlled groups
Full -text articles that are published in the English language and have an abstract	Articles that were published before 2012
Articles that compare NEXUS criteria and other decision support techniques for pediatric patients with spinal trauma or CSI	Non-English articles
	Articles without abstract and or full text

Outcome measures

The primary objective is to determine the accuracy of the NEXUS criteria when evaluating CSI and other spinal trauma in pediatric patients. The diagnostic performance of the NEXUS criteria was the main outcome evaluated in the study. The other key outcome that was evaluated is the sensitivity reported in the included studies. The number of cases that were missed when applying the NEXUS criteria was recorded and used to compute the sensitivity of the technique in the studies where it was not explicitly expressed.

Quality evaluation of eligible articles

The methodological quality of the included studies was evaluated using the Jadad score. The studies are assessed in three areas; randomization, blinding, and accountability. The first five items scored 0 (No) and 1 (Yes). The last two items scored 0 (No) and -1 (Yes). Table 2 shows a summary of the five items that are assessed under the Jadad score. Studies with a score between 3 and 5 were considered high quality, and studies between 0 and 2 were considered low quality [15].

**Table 2:** Jadad score items (Berger and Alperson, 2009) [15]

Question	Description	No/Yes
Q1	The study is described as randomized	0/1
Q2	The randomization approach is described and appropriate	0/1
Q3	The study described as double-blind	0/1
Q4	The double-blinding method is described and appropriate	0/1
Q5	There is a description of withdrawals and dropouts	0/1
Q6	The method used in randomization was described, and it was inappropriate	0/−1
Q7	The method of blinding was described, and it was inappropriate	0/−1

Data extraction and analysis

The data collected from the extraction process include the study ID (author and year of publication), participants, study design, clinical parameters, reference standard, primary and secondary outcomes, and results. The data on the primary and secondary outcomes of the included studies were also extracted, summarized, and tabulated. The literature review did not apply any quantitative data analysis to the collected data. The differences in the age groups of adolescents and other methodological inconsistencies implied that pooling and analysis of quantitative findings were not possible. The methodological analysis of the included studies justifies the lack of quantitative analysis.

Results

The researcher identified 1657 articles after applying the search strategy in the databases and 56 from reference lists of primary studies and systematic analyses. Of the identified articles, 849 were excluded due to duplication. An additional 306 were excluded from the topic and abstract screening stages for lack of relevance to the research topic. The remaining articles were considered for retrieval; 111 articles were then excluded due to a lack of full-text copies.

**Figure 1:**
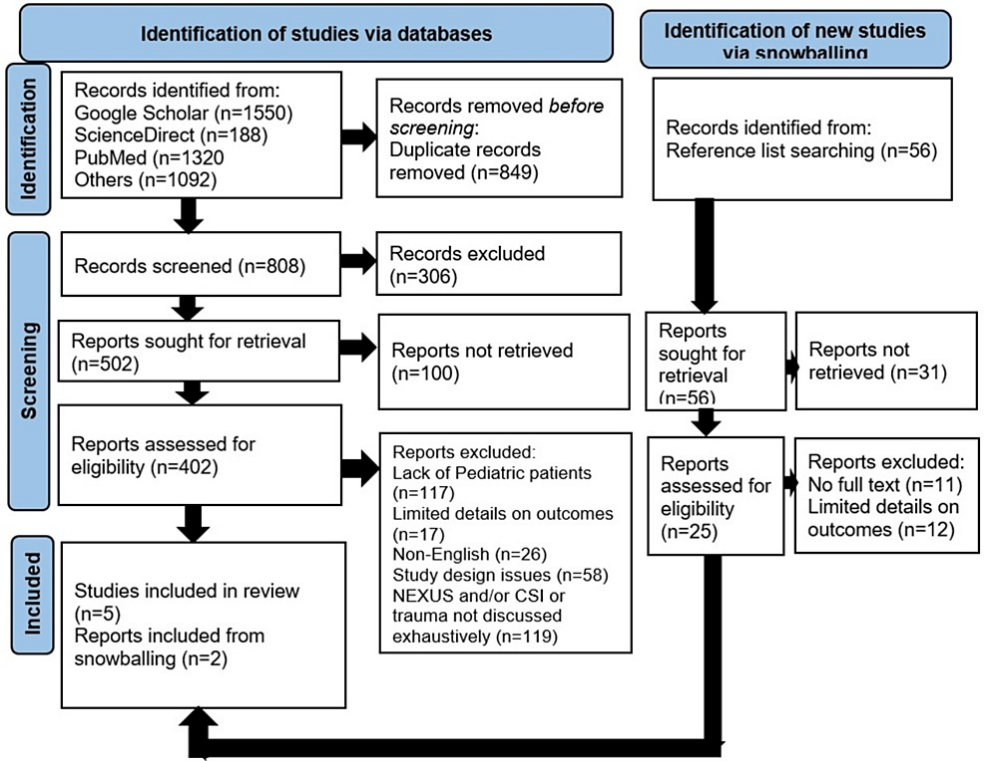
Preferred Reporting Items for Systematic Reviews and Meta-Analyses (PRISMA) flow diagram outlining the included studies

A total of 144 articles were further excluded due to issues with the sample and missing outcomes. The researcher also excluded 26 non-English studies and another 58 due to non-experimental study designs (commentaries, literature reviews, and opinion pieces). Of the remaining articles, 208 were excluded for failing to meet other inclusion criteria. Finally, the researcher retrieved seven articles for summary and analysis. Figure 1 shows the Preferred Reporting Items for Systematic Reviews and Meta-Analyses (PRISMA) diagram depicting the identification, screening, and inclusion processes used in the literature review.

Table 3 below outlines the characteristics of the included studies. Results of each of the studies utilizing NEXUS criteria are also examined as part of this literature review

**Table 3:** Summary of evaluated studies with outline of outcome measures NLC: NEXUS low risk criteria; CCR: Canadian C-Spine (cervical-spine) Rule; NEXUS: National Emergency X-Radiography Utilization Study; CT: Computed Tomography; MR: Magnetic Resonance

Author	Year	Number of participants (N)	Study Design	Clinical Parameters	Reference Standard	Outcomes Measured	Results
Ehrlich et al. [16]	2009	NEXUS (108) Canadian C-Spine (cervical spine) Rule 109	Retrospective	NEXUS/NLC Canadian C-Spine (cervical spine) Rule (CCR)	Plain C-Spine radiographs, CT scans, or both	Missed injuries, Injury severity score, ability to apply the CCR or NLC criteria, and reduction or increased requirements for decision rules per cohort	CCR or NEXUS criteria are not sensitive or specific enough
Anderson et al. [17]	2006	583	Prospective	NEXUS	CT and MR imaging	Cervical spine cleared, late injuries found, op stabilization, dislocation, fracture, ligamentous injury only	NEXUS was important in detecting cervical spine injuries in pediatrics and increase the number of cervical spines cleared
Overmann et al. [18]	2019	Hypothetical 10 years old incorporating parameters for patients aged 0-17 years	Case study	NEXUS	CT scan screening cervical radiographs	Sensitivity, specific, quality of life, costs, and life expectancies for the 10 years old base case	Using a NEXUS first to assess risk before diagnostic testing is less costly and more effective than performing CT scanning or cervical screening radiographs
Rosati et al. [5]	2015	233	Retrospective	NEXUS	CT scan, cervical spine plain films/MRI	Injury severity score, clearance by NEXUS criteria	NEXUS implementation decreases pediatric cervical spine CT usage and should improve across time
Viccellio et al. [11]	2001	3065	Retrospective	NEXUS	Cervical CT or MRI of the entire cervical spine	Sensitivity, specificity, injury rate, the prevalence of individual low-risk criteria, number of low-risk patients, number of low-risk patients with injury	NEXUS is cost effective

Table 4 summarizes the factors and Jadad score of the included to assess the methodological qualities of the included studies

**Table 4:** Factors and Jadad score

Author	Year	Q1	Q2	Q3	Q4	Q5	Q6	Q7	Total Jadad Score
Ehrlich et al. [16]	2009	1	1	1	1	0	0	0	4
Anderson et al. [17]	2006	0	0	0	0	0	0	0	0
Overmann et al. [18]	2019	0	0	0	0	0	0	0	0
Viccellio et al. [11]	2001	0	0	0	0	0	0	0	0

Quality of study analysis

Based on the Jadad scores for the studies, only one could be considered a high-quality source. Other than failing to describe the withdrawals, the study by Ehrlich et al. [16] specified the randomization and blinding processes that were used. In all studies, points were not deducted for questions 6 and 7 because randomization and blinding were either not described or appropriate when described. The findings demonstrate that the studies cannot be used to generate accurate quantitative analysis by pooling an individual source's findings.

Nature of included studies

There were a total of 4502 pediatric patients in the included studies. The number of patients in the studies ranged from a minimum of 1 and a maximum of 3065. While six studies only looked at the NEXUS criteria, Ehrlich et al. [16] compared NEXUS and the Canadian C-Spine Rule (CCR). Five of the studies were retrospective, while one by Anderson et al. [17] was prospective and another by Overmann et al. [18] was a case study. The reference standards used in the studies include CT scan (7/7, 100%), MRI (4/7, 57%), X-ray (2/7, 29%), and cervical spine radiographs (2/7, 29%). In one study Ehrlich et al. [16] simultaneously used two reference standards for some patients.

Impact of NEXUS criteria

The study by Rosati et al. [5] found that 18% received a cervical spine CT in the instances where the NEXUS criteria were not applied, while the proportion increased to 61% in the instances where the NEXUS criteria were applied. After 12 months, the number of CT scans performed on pediatric patients decreased by 23% (p=0.01) when the NEXUS criteria were applied. The researchers also reported a 16% (p=0.01) when standards other than CT (MRI and plain films) were used. These changes were observed despite the lack of significant difference in age and injury severity across the two years.

In their study, Overmann et al. [18] focused on the cost-effectiveness of the NEXUS criteria when used with X-rays and/or CT. The researchers found that risk stratifying pediatric patients based on the NEXUS criteria before selectively using X-ray and CT was cost effective.

Sensitivity of NEXUS criteria

Four of the included studies measured the sensitivity of the NEXUS criteria. Overmann et al. [18] reported a sensitivity of 0.99(0.87-1) in pediatric patients when the NEXUS criteria were applied. Another study by Viccellio et al. [11] found 100% sensitivity with a range of 87.8%-100.0% for pediatric patients compared to 99.02% (98.0%-99.6%) for the overall sample that included adults. Ehrlich et al. [16] compared the sensitivity of NEXUS and CCR. The sensitivity of NEXUS was 43%, while the sensitivity of CCR was 86%. The confidence level in all of the included studies examining sensitivity is 95%.

Specificity of NEXUS criteria

Three studies measured the specificity of the NEXUS criteria. Overmann et al. [18] reported a specificity of 0.54(0.19-0.64), and Ehrlich et al. [16] found that the specificity of NEXUS/NLC was 96%, while the specificity of CCR was 94%. Viccellio et al. [11] found 19.9% specificity with a range of 18.5%-21.3% for pediatric patients compared to 12.93% for the overall sample that included adults. The confidence level in all of the included studies is 95%.

Potential of clearing CSI with NEXUS criteria without radiographical examination

Three studies by Ehrlich et al. [16], Anderson et al. [17], and Viccellio et al. [11] examined the potential of using the NEXUS criteria in clinically clearing CSI in pediatric patients. Ehrlich et al. [16] retrogressively examined two groups of pediatric patients 10 years or younger and performed cervical spine imaging in one group and no imaging in the second before applying NEXUS and CCR. The researchers found that the sensitivity and specificity of NEXUS were 43% and 96%, respectively. They also found that the sensitivity of CCR was 86%, while specificity was 94%. Based on the findings, there was not much to separate the two rules on specificity despite CCR having higher sensitivity than NEXUS. The researchers concluded that both criteria lacked the specificity and sensitivity that qualify them as ideal standards for cervical spine imaging in pediatric patients.

Anderson et al. [17] explored the possibility of using the criteria without imaging. The researchers compared rates of cervical spine clearance using the NEXUS criteria versus consulting neurosurgical personnel. In the group to which the NEXUS criteria were applied, 62.4% were cleared, while the neurosurgical personnel cleared 95% of the patients enrolled without the protocol. Despite being no late injuries in the NEXUS and non-NEXUS groups, the difference in clearing rate was significant. The researchers, however, recognized the limitation of applying the NEXUS criteria after obtaining cervical spine radiographs. The prospective multicenter study by Viccellio et al. [11] also attempted to examine the reliability of the NEXUS criteria for clearing the cervical spine clinically for patients under 18 years. The researchers found that 19.7% of the participants met the low-risk criteria. Additionally, the researchers found that none of the cleared patients had CSI. The results demonstrated a 100% sensitivity.

Discussion

The findings show that the NEXUS criteria have the potential to be used in pediatric patients with CSI and spinal trauma. However, the results of the present literature review show that despite the potential value of the NEXUS criteria, the use of the decision guideline has not been validated in the current literature for CSI clearance in pediatric patients. The reviewed studies show that the sensitivity and specificity of the NEXUS criteria are inadequate. In their studies, Ehrlich et al. [16], Anderson et al. [17], and Viccellio et al. [11] explored if pediatric cervical spine patients could be cleared without radiographic examination and concluded that the NEXUS criteria could be cautiously applied for this purpose [11,16,17]. Overmann et al. [18] identified the cost benefit as a key value of using the NEXUS criteria for pediatric patients.

It is important to understand the nuanced differences between pediatric and adult patients, and their impact on the sensitivity and specificity of the NEXUS criteria. The study by Ehrlich et al. [16] narrowed down to patients aged below 10 years old since they had not attained puberty. They further found anatomical differences in children younger than 8 years old with more rostral injuries than older children. Current evidence demonstrates prominent anatomical differences between the cervical spines of adults and children under 8 years old; those differences largely disappear during growth from 8-12 years of age [19]. The researchers concluded that the specificity and sensitivity of their study would have been significantly lower if the sample had more patients aged under 8 years. There is a possibility that these anatomical differences account for the lower sensitivity and specificity of the NEXUS criteria in pediatric patients. These effects are expected to be worse when the criteria are applied to infants and toddlers.

The sensitivity of the NEXUS criteria in the included studies is consistent with other adult studies. Other than one study, the rest of the studies reported that the sensitivity was below the 99.6% reported in the original NEXUS study. In their study, Dickinson et al. [20] reported that the sensitivity of the NEXUS criteria was 92.7%. These findings raised some doubt about the validity of the original study.

Other than the anatomical differences, the lower sensitivity and specificity can also be attributed to the inability of younger children to verbalize how they are feeling. Therefore, nonverbal and unconscious children must be cautiously treated using the NEXUS criteria [21]. In their study of pediatric patients with head trauma, Laham et al. [22] noted that children under the age of two years are nonverbal and thus high-risk. Therefore, the stratification of pediatric patients as high-risk (<2 years) and low-risk (verbal, >2 years) is necessary for determining the validity of the NEXUS criteria in the group. According to Kreykes and Letton [23], adolescents and teens are cooperative enough to be clinically assessed using the NEXUS criteria with a sensitivity similar to adults, proving this point. These findings show that pediatric patients need to be stratified when determining the validity of the NEXUS criteria rather than fitting all children in one group. Additionally, they show the limitations of NEXUS in assessing infants and children and the potential value of using other decision tools, such as the American Association for the Surgery of Trauma (AAST) [23].

Despite the value of the included studies in providing insights on the effectiveness of the NEXUS criteria in determining the need for imaging in pediatric patients with CSI, the limited amount of literature on the topic remains a concern. In addition, the inconsistencies in sensitivity and specificity can also be attributed to the differences in methodology used in these studies. For example, while Anderson et al. [17] demonstrated that the rate of clearing the cervical spine without the NEXUS criteria is high, the study is limited in its use of radiographs of the cervical spine [17]. The persistence of these issues implies that it will be difficult to determine the validity of applying the NEXUS criteria in pediatric patients when determining the need for cervical spine imaging.

Overmann et al. [18] demonstrated that applying the NEXUS criteria is critical in promoting cost savings in the care of children. The cost saving is a component of the reduced probability of radiation-induced malignancy due to exposure to cervical spine CT. Current evidence shows that the increased lifetime risk per unit dose and increased dose per milliampere-second in children compared to in adults lead to increased medical risks and costs of pediatric CT [24]. With the malignancy risk significantly increasing in children compared to adults, there is a strong case for using the NEXUS criteria for pediatric patients, given its potential to reduce exposure to large amounts of radiation from CT.

Comparing the accuracy of NEXUS and other diagnostic standards is important in determining its potential for pediatric cervical spine trauma patients. The retrogressive study by Ehrlich et al. [16] compared the NEXUS criteria with CCR. While previous studies have attempted to compare the two standards, they have largely focused on adult or mixed populations. Adult studies have demonstrated that the NEXUS criteria are outperformed by other diagnostic guidelines. In their systematic review of adult patients, Michaleff et al. [25] found that the CCR had better diagnostic accuracy than the NEXUS criteria. Stiell et al. also found that the CCR is more sensitive than the NEXUS criteria (99.4% versus 90.7%, p<0.001). In addition, the researchers found that the NEXUS criteria missed injuries in 16 patients while CCR only missed injuries in one patient [26]. While the study by Ehrlich et al. [16] could not fully address the methodologic issues highlighted by Michaleff, it focused on the pediatric population. Unlike Michaleff et al. [25], Ehrlich et al. [16] found that neither NEXUS nor the CCR had adequate specificity or sensitivity to be preferred for pediatric patients. Dickinson et al. also concluded that neither NEXUS nor CCR could be conclusively determined as the most effective and efficient decision rule [20]. Despite the non-committal conclusions by the researchers, there is a consensus that NEXUS and CCR are important in reducing the inefficient use of cervical spine radiography given the low sensitivity. For instance, Dickinson et al. reported a 6.1% reduction in cervical spine radiography rates (from 68.9 to 62.8%). Despite the reduction in the sensitivity relative to the original study, the value of the NEXUS criteria in improving decision making is evident in the included studies.

The differences in performance between CCR and the NEXUS criteria can be understood by looking at other comparative analysis studies. Stiell et al. argue that adding the fifth criterion (no focal deficit) without adequate empirical support is one factor that explains the NEXUS criteria's low sensitivity [26]. Additionally, the inclusion of infants and children in their NEXUS criteria group is another reason for the low sensitivity reported in their study. Finally, the potential of familiarity with or bias toward the CCR might explain why it performed better than the NEXUS criteria. When examined along with the low sensitivity of the other studies included in this review, there is a legitimate reason for increasing the use of the NEXUS criteria in the pediatric environment

One of the key attributes of using the NEXUS criteria is eliminating the need for lateral neck radiographs in trauma patients [27]. However, the poor performance of NEXUS compared to other decision rules such as the New Orleans Criteria (NOC) and the Canadian CT Head Rule (CCHR) in assessing the need for radiology in pediatric head trauma also demonstrates that there is reason to doubt its wider use [28]. Given the potential value that NEXUS and other decision rules have on the course of treatment, the accuracy of the tools must be improved before being considered the standard for pediatric cervical spinal evaluation.

One of the criticisms that have been leveled against the NEXUS criteria is that they are vague and poorly reproducible [29,30]. Given the results of the included studies, this is especially the case when the criteria are applied to pediatrics. The knowledge gap in using NEXUS and other guidelines for pediatric patients is another potential reason for the low sensitivity reported in the included studies. In their study, Burns and Yanchar found that 32% of physicians did not use any guidelines, with 85% noting that they would use them if available [31]. Healthcare organizations are obligated to provide guidelines and empower physicians to use these tools to improve the evaluation of pediatric patients for cervical spinal imaging. Currently, most institutions have a low adoption of cervical spine clearance protocols (46%) as most use CT, X-ray, or a combination as the primary imaging modality [32].

While most of the included studies lacked controls, Rosati et al. [5] compared the changes in the number of CT scans cleared by the NEXUS criteria in one period to another after 12 months. In the initial 12-month period where guidelines were not implemented, the researchers found that only 18% of patients received CT, compared to 61% who were cleared by the NEXUS criteria [5]. The findings show that the NEXUS criteria were useful in capturing the cases that might have been missed otherwise. In the following 12-month period, the researchers found that the CT scan clearable by the NEXUS criteria decreased by 23% (p=0.01) when the guideline was implemented. The study is important as it demonstrates the value of implementing a guideline while applying the NEXUS criteria. The researchers also reported similar rates in the non-CT cases where MRI and plain films are used. The findings demonstrate that the use of the guidelines improves over time and reduces the proportion of patients approved for imaging.

The lack of consensus on the validity of the NEXUS criteria for children leaves its application in question. The wide confidence intervals in the specificity and sensitivity of the NEXUS criteria are a legitimate concern in the validation of its application in pediatric patients [18]. Garton and Hammer [33] also measured the sensitivity of the NEXUS criteria in spinal trauma assessment. Among the children younger than 8 years, the researchers found a sensitivity of 94%, with two cases missed out of 34 patients. For the 158 children aged over 8 years, the researchers reported 100% sensitivity as all injuries were reported based on the NEXUS criteria [33]. The current literature review shows no consensus on the validity of the NEXUS criteria in children. Multiple potential reasons have been presented to explain the poor performance of NEXUS in pediatric patients relative to adults. The strategies used in adult trauma do not always translate to children due to limitations inherent to their age, as well as the possibility of mental state alteration due to head injury [23,33].

Strengths and limitations

One of the strengths of the present study is the use of multiple databases and reference list searches to identify potential studies. In addition, applying robust search criteria ensured that only eligible articles were selected. The other strength of the study is the focus on pediatric patients. This adds to the current literature largely focused on adult populations.

The limited number of studies that qualified for inclusion is one of the current study's limitations. The limited studies on CSI and spinal trauma and the application of the NEXUS criteria for what is a rare condition in pediatric patients account for this limitation. The studies that are included in this systematic analysis have some limitations themselves. The low number of participants in some of the studies contributes to the current study's limitations.

The lack of a quantitative component that pools the results of the studies implies that the significance of the findings cannot be pooled and integrated to gain a comprehensive understanding of applying the NEXUS criteria to pediatric patients. Future studies need to have a meta-analysis component to better understand the application of the NEXUS criteria in this population. In addition, the methodologies of the included studies presented a challenge. The lack of randomized controlled trials (RCTs) is a limitation of the current review. Prospective studies that allow for the criteria to be assessed as the physician is determining the need for imaging are important in getting more accurate results on the effectiveness of the NEXUS criteria in pediatric patients. The lack of controls is another concern, with only one study comparing NEXUS with another decision guideline.

Finally, the present review is limited as only one study was considered high quality based on the Jadad score. In addition, the absence of elements such as randomization and blinding implies that most of the included studies have some concerns that might limit their reliability in addressing the topic. Future studies need to address these limitations better to understand the application of the NEXUS criteria in pediatric patients.

## Conclusions

While rarely encountered in the pediatric population, CSI can have significant consequences when missed. This emphasizes the need for a reliable decision rule to more precisely identify such injuries in the pediatric population. The results of this systematic review demonstrate that the support for applying the NEXUS criteria in determining the need for cervical spine imaging in pediatrics is weak. Based on the included studies, the NEXUS criteria has been shown to have low sensitivity, reliability, and clinical acceptability. Therefore, the researchers are largely cautious about recommending using the NEXUS criteria as the preferred standard. Despite these concerns, there is evidence of increased effectiveness and cost-effectiveness in using the NEXUS criteria in pediatric patients.

The findings from the included studies present practice and research implications that need to be considered to get the most out of using the NEXUS criteria. From a practice perspective, the findings demonstrate that the NEXUS criteria can be used to improve decision-making on cervical spine imaging of pediatric patients. The application of the NEXUS criteria is not only useful in avoiding the cost of unnecessary CT imaging, but also the exposure of pediatric patients to large amounts of radiation inherent to the modality. Healthcare institutions are responsible for increasing the use of guidance protocols for pediatric patients. While CSI might be rare among pediatric patients, the significant risk of morbidity makes it important to understand the effectiveness of assessment approaches. The potential of the NEXUS criteria to be applied to this group and minimize radiation exposure makes it critical to evaluate its value. There is a need for future studies to focus on and stratify pediatric patients and use prospective designs to gain a better understanding of the validity of applying the NEXUS criteria to pediatric patients.
